# Prenatal and Early, but Not Late, Postnatal Exposure of Mice to Sidestream Tobacco Smoke Increases Airway Hyperresponsiveness Later in Life

**DOI:** 10.1289/ehp.0800511

**Published:** 2009-05-22

**Authors:** Zhong-Xin Wu, Dawn D. Hunter, Vincent L. Kish, Katherine M. Benders, Thomas P. Batchelor, Richard D. Dey

**Affiliations:** 1 Department of Neurobiology and Anatomy and; 2 Department of Orthopedics, Robert C. Byrd Health Sciences Center, West Virginia University, Morgantown, West Virginia, USA

**Keywords:** airway innervation, asthma, muscarinic agonists, neurokinin receptor, neurotrophic factor

## Abstract

**Background:**

Cigarette smoke exposure *in utero* and during early postnatal development increases the incidence of asthma and airway hyperresponsiveness (AHR) later in life, suggesting that a possible critical period of developmental sensitivity exists in the prenatal and early postnatal periods.

**Objective:**

We investigated mechanisms of susceptibility during critical developmental periods to sidestream smoke (SS) exposure and evaluated the possible effects of SS on neural responses.

**Methods:**

We exposed three different age groups of mice to either SS or filtered air (FA) for 10 consecutive days beginning on gestation day (GD) 7 by maternal exposure or beginning on postnatal day (PND) 2 or PND21 by direct inhalation. Lung function, airway substance P (SP) innervation, and nerve growth factor (NGF) levels in broncho alveolar lavage fluid were measured after a single SS exposure on PND59.

**Results:**

Methacholine (MCh) dose response for lung resistance (R_L_) was significantly elevated, and dynamic pulmonary compliance (C_dyn_) was significantly decreased, in the GD7 and PND2 SS exposure groups compared with the FA groups after SS exposure on PND59. At the same time points, the percent area of SP nerve fibers in tracheal smooth muscle and the levels of NGF were significantly elevated. MCh dose–response curves for R_L_ and C_dyn_, SP nerve fiber density, and the level of NGF were not significantly changed in the PND21 exposure group after SS exposure on PND59.

**Conclusions:**

These results suggest that a critical period of susceptibility to SS exposure exists in the prenatal and early postnatal period of development in mice that results in increased SP innervation, increased NGF levels in the airway, and enhanced MCh AHR later in life.

Environmental tobacco smoke (ETS) is an environmental trigger that leads to airway inflammation and asthma symptoms in susceptible individuals and animals (Martinez 2003; Weiss 1994, 1999). Exposure to ETS *in utero* or during early postnatal development increases the incidence of respiratory illnesses (Gilliland et al. 2002; Haberg et al. 2007; Li et al. 2005; Martinez et al. 1992; Raherison et al. 2007; Wang et al. 2008; Weitzman et al. 1990) and airway hyperreactivity (Frischer et al. 1992; Skorge et al. 2005; Tager et al. 1983) later in life. The nervous system, including the nerves supplying the airways, is highly susceptible to environmental influences during development. Epidemiologic studies show that the probability of developing or exacerbating childhood asthma is increased in children of mothers who smoke cigarettes (Gilliland et al. 2002; Haberg et al. 2007; Li et al. 2005; Martinez et al. 1992; Raherison et al. 2007; Wang et al. 2008; Weitzman et al. 1990). These results suggest a possible critical period of developmental sensitivity to cigarette smoke exposure during the prenatal and early postnatal period.

Airway innervation develops rapidly during fetal and early postnatal life, in parallel with the development of the lungs (Dey and Hung 1998). Given the dynamic and vulnerable nature of developmental processes, this period of morpho genesis is likely to be exquisitely sensitive to environmental insults (Dietert et al. 2000; Pinkerton and Joad 2000). Substance P (SP), a neuropeptide and member of the tachykinin family, has potent effects on airway smooth muscle tone, vascular permeability, and mucus secretion (Barnes et al. 1991; Lundberg et al. 1983b, 1984). SP plays an important role in antigen- or irritant-induced airway hyperresponsiveness (AHR) and asthma (Ollerenshaw et al. 1991; Wu and Lee 1999; Wu et al. 1997, 2000, 2001). Our research has demonstrated that increased SP levels in the airway are involved in cigarette-smoke exposure–enhanced airway constriction to methacholine (MCh) (Wu and Lee 1999).

Nerve growth factor (NGF) is a neurotrophic factor that promotes and maintains growth and survival of the central and peripheral nervous system (Levi-Montalcini 1987). NGF in the lung increases during gestation and decreases progressively with postnatal age (Hu et al. 2002). Disruption of normal synthesis and release of NGF results in changes in airway innervation that lead to disease-related abnormalities in the respiratory system (Hu et al. 2002; Tortorolo et al 2005; Wilfong and Dey 2004, 2005). Our recent studies have shown that NGF is produced in response to irritant exposures and mediates changes in the phenotype and distribution of SP-containing neurons in the airways (Wilfong and Dey 2004, 2005).

Thus, we hypothesized that susceptibility to ETS exposure exists during prenatal and early postnatal periods, and that ETS exposure during these “critical periods” would alter SP airway innervation, possibly through an NGF-mediated mechanism, increasing susceptibility to AHR in later life. We designed the present experiments to identify critical developmental periods of susceptibility to sidestream smoke (SS; a surrogate for ETS exposure) in mice and to characterize changes in SP innervation and NGF expression during critical periods of developmental susceptibility to SS.

## Materials and Methods

ICR mice (Harlan, Indianapolis, IN) were housed with *ad libitum* access to food and water in a facility approved by the U.S. Food and Drug Administration. All procedures were performed in accordance with the *Guide for the Care and Use of Laboratory Animals* (Institute of Laboratory Animal Resources 1996), and the protocols were approved by the West Virginia University Animal Care and Use Committee (protocol 06–0501). The animals were treated humanely and with regard for alleviation of suffering.

### SS exposure

**Figure 1:**
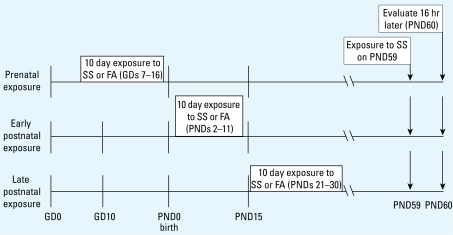
Time line for SS exposure and experimental measurement. Prenatal exposures occurred between GD7 and GD16; early postnatal exposure occurred PNDs 2–11; late postnatal exposure occurred PNDs 21–30. All groups were exposed to SS on PND59. Pulmonary function testing was conducted immediately before and 16 hr after reexposure. Samples for all other evaluations (bronchoalveolar lavage and airway removal for immunocytochemistry) were collected 16 hr after exposure.

We investigated the effects of SS exposure in three different ages of mice: gestation days (GDs) 7–16, which corresponds to the period of human fetal lung development; postnatal days (PNDs) 2–11, which corresponds to the human neonatal period; and PNDs 21–30, which is similar to the human prepubertal time period (Pinkerton and Joad 2000; Wang and Pinkerton 2008). Tobacco smoke exposure in monkey, rat, or mouse models has shown that ETS exposure for 6 hr/day for a few weeks to 2 months alters pulmonary function and immune responses (Barrett et al. 2002; Ji et al. 1998; Joad et al. 1995; Yu et al. 2008). We exposed mice in each age group to either SS or filtered air (FA) for 6 hr/day for 10 consecutive days beginning on GD7 (by maternal exposure) or on PND2 or PND21 (direct exposure; Figure 1). The 10-day exposure periods were chosen to allow testing of our experimental design in nonoverlapping developmental time periods. In addition, by 2 weeks of age, mouse lungs and the pulmonary nervous systems are nearing maturity; thus, exposure in the PND2 group was completed before lung maturity and exposure in the PND21 began after maturity. For the GD7 group, exposure began 10 days before the approximate date of parturition, during the period when lung buds are beginning to form and nerves begin to invade the lung (Tollet et al. 2002).

**Figure 2:**
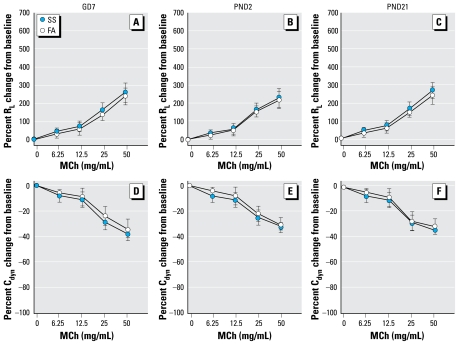
MCh dose responses of R_L_ and C_dyn_ in GD7 (*A* and *D*), PND2 (*B* and *E*), and PND21 (*C* and *F*) FA- or SS-exposed mice before SS exposure on PND59. Data are mean ± SE of 6 mice/group.

**Figure 3:**
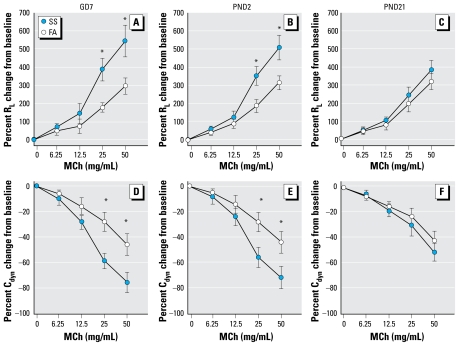
MCh dose responses of R_L_ and C_dyn_ in GD7 (*A* and *D*), PND2 (*B* and *E*), and PND21 (*C* and *F*) FA- or SS-exposed mice after SS exposure on PND59. Data are mean ± SE of six mice/group. *Significant difference in corresponding data between FA and SS animals (*p ≤* 0.05).

Pulmonary function testing was measured before or 16 hr after a 6-hr exposure to SS on PND59 (Figure 1). The intent of this design was to examine plasticity of airway responsiveness and potential mechanisms associated with early-life exposure by comparing responsiveness to an irritant exposure (SS) after a period of recovery in naive mice and mice exposed to SS at three stages of early life. Because we found that pulmonary function was altered only after exposure to SS on PND59 (Figures 2 and 3), SP nerve fiber density (NFD) and NGF and inflammatory cells in bronchoalveolar lavage fluid (BALF) were measured only after the PND59 exposure.

By classical definition, ETS is a diluted mixture of the smoke given off by the burning end of a tobacco product (SS, ~ 85%) and the smoke exhaled by smokers (mainstream smoke, ~ 15%). Base on previous ETS exposure studies (Pinkerton and Joad 2000; Yu et al. 2008), we used SS as a surrogate for ETS. The SS exposure protocol in this study used the same exposure equipment and followed the methods described previously (Porter et al. 2007). Briefly, randomly chosen mice were placed in a BioClean exposure chamber (DuoFlo, model H 5500; Lab Products Inc., Maywood NJ) that measured 1.92 × 1.92 × 0.97 m (3.58 m^3^). The mice were housed in separate cages located inside the exposure chamber. SS from Marlboro filter cigarettes (Phillip Morris, Richmond, VA) was introduced into the exposure chamber at a rate of four cigarettes every 15 min for 6 hr/day for 10 days using a smoking machine (RM 1/G; Heinr Borgwald GmbH, Hamburg, Germany). At the end of the 6-hr exposure period, the exhaust fan on the BioClean unit was turned on to rapidly lower the level of SS in the exposure chamber. The mice were then transported to the animal facilities, where they remained overnight, until the next day’s exposure. The concentrations of carbon monoxide in the exposure chamber were monitored and kept to an average of about 50 ppm; relative humidity was about 50%, and temperature was approximately 23°C. Total suspended particulate concentration was approximately 1.1 mg/m^3^, similar to exposure levels used by others to approximate the cloud of particulates surrounding a person during active smoking (Yu et al. 2008). The level of nicotine in blood was also measured immediately after 1 week of SS or FA exposure. Briefly, blood was drawn from the orbital plexus of each mouse (eight mice for SS and eight mice for FA) and allowed to clot at room temperature for 30–45 min. The resulting serum was stored at −80°C and was assessed by an independent laboratory (National Medical Services, Willow Grove, PA) using gas chromatography.

### Measurements of lung function

We determined lung function by measuring changes of pulmonary resistance (R_L_) and dynamic compliance (C_dyn_) after aerosolized MCh challenge using a modification of our previously described technique (Kwong et al. 2001; Wu and Lee 1999; Wu et al. 1997, 2000). Briefly, mice were anesthetized with pentobarbital (70 mg/kg, intraperitoneally) before or 16 hr after exposure to SS or FA on PND59. The trachea was cannulated just below the larynx via a tracheotomy, and a four-way connector was attached to the tracheotomy tube. Two ports were connected to the inspiratory and expiratory tubes of a respirator (model 845; Harvard Instruments, Holliston, MA). The mice were ventilated at a constant rate of 200 breaths/min and a tidal volume of approximately 0.2 mL. Aerosolized MCh chloride (Sigma, St. Louis, MO) was administered for 10 sec in increasing concentrations (0, 6.25, 12.5, 25, and 50 mg/mL). For 5 min before and after each MCh challenge, total R_L_ and C_dyn_ were analyzed by computer on a breath-by-breath basis.

### Inflammatory cell analysis in BALF

We obtained BALF by injecting and withdrawing 1 mL of sterile saline three times (3 mL total) via the tracheal cannula 16 hr after exposure to SS on PND59. The collected BALF (~ 2.5 mL) was centrifuged at 1,500 rpm for 10 min. The supernatant was frozen at −80°C for NGF assays, and the pelleted cells were treated with Tris-buffered ammonium chloride solution (pH 7.2) to lyse red blood cells. The remaining cells were washed once with phosphate-buffered saline (PBS) supplemented with 1% fetal calf serum, 100 U/mL penicillin, 100 μg/ mL streptomycin, and 250 ng/mL amphot-ericin (Gibco Brl, Grand Island, NY). Total cell counts were determined using a hemo-cytometer. Differential leukocyte counts were then performed on cytospin slides. A minimum of 200 leukocytes and macrophages were counted using standard morphologic criteria.

### NGF enzyme-linked immunosorbent assay (ELISA)

We conducted the NGF ELISA as previously described by Wilfong and Dey (2004, 2005). Briefly, the BALF supernatants collected 16 hr after exposure to SS on PND59 were frozen at −80°C until assayed. The concentration of NGF (7.8–500 pg/mL) in each sample was assayed using the NGF Emax ImmunoAssay System (Promega, Madison, WI) according to the manufacturer’s instructions. NGF was detected using an antibody sandwich format in 96-well plates. Each well was initially coated with 100 μL of anti-NGF antibody and incubated overnight, followed by a 1-hr incubation with blocking buffer (200 μL/well) to prevent nonspecific binding. Either 100 μL lavage supernatant or 100 μL NGF standard (7.8–500 pg/mL) was then added to each well. The plate was incubated for 6 hr, followed by an overnight incubation with anti-NGF monoclonal antibody (100 μL/well). For color development, an anti-rat IgG-horseradish peroxidase–conjugated antibody was added to each well (100 μL), followed by a tetramethlybenzidine solution that reacted with the peroxidase-labeled conjugates to develop a blue color. The absorbance of each well was measured at 450 nm on a Spectra Max 340pc plate reader (Molecular Devices, Sunnyvale, CA). The concentration of NGF in each lavage sample was determined from the NGF standard curve. All samples were run in duplicate or triplicate, and a PBS sample was run with each assay as a negative control.

### Immunocytochemistry

The procedures for immunocytochemical demonstration of SP-like immunoreactivity have been described previously (Dey et al. 1999; Wu et al. 2002, 2004). Briefly, tracheal segments were removed 16 hr after exposure to SS on PND59, fixed in picric acid/formaldehyde fixative for 3 hr, and then rinsed three times with 0.1 M PBS containing 0.3% Triton X-100 (PBS-TX), frozen in isopentane, cooled with liquid nitrogen, and stored at −80°C. Cryostat sections (12 μm thickness) were collected on gelatin-coated cover slips and dried briefly at room temperature. Sections were covered with SP antibody (diluted 1:200; Peninsula Inc., Belmont, CA), incubated at 4°C for 24 min, rinsed three times with a 1% bovine serum albumin/ PBS-TX solution, covered with fluorescein isothiocyanate–labeled goat anti-rabbit antibody (diluted 1:100; ICN Immunobiologicals, Costa Mesa, CA), incubated at 37°C for 30 min, and rinsed. After all immunocyto-chemical procedures were conducted, the cover slips were mounted with Fluoromount and observed with a fluorescence microscope.

For measuring NFD in tracheal smooth muscle, we collected images of SP-containing nerve fibers in series with a Zeiss LSM 510 confocal microscope (Carl Zeiss MicroImaging GmbH, Göttingen, Germany). A series of images representing all of the tracheal smooth muscle in a section were collected in digital files, saved to an internal database, and measured digitally with Optimas software (Media Cybernetics Inc., Bethesda, MD). The smooth muscle regions were outlined to measure total cross-sectional area of smooth muscle. SP-positive nerve fibers were identified by segmentation using threshold gray levels with the Optimas software. NFD was then calculated as percentage of SP-immunoreactive nerve fiber area based on the total cross-sectional area of smooth muscle. At least 10 measurements were made for each section, and eight sections were measured in each animal.

### Data analysis

Unless otherwise stated, results are expressed as mean ± SE. Values for R_L_ and C_dyn_ elicited by MCh are expressed as a percentage of the baseline. NFD is expressed as percent area of SP-immunoreactive nerve fibers in the total area of the smooth muscle. Statistical analyses of lung function, inflammatory cell, and NGF release in BALF were performed using one-way or two-way analysis of variance. A *p*-value < 0.05 was considered significant, and *n* represents the number of animals studied.

## Results

### Effect of SS during prenatal or postnatal period on the changes of lung function

The average nicotine concentration in serum was about 0 ng/mL after FA exposure; nicotine measured in the SS-exposed animals at the end of the SS exposure was approximately 20 ng/mL [95% confidence interval (CI), 15.5–24.5; *n* = 8], which is similar to nicotine levels typically found in human smokers (10–50 ng/mL) (Benowitz and Jacob 1984; World Health Organization 2003).

The baseline R_L_ and C_dyn_ values in the different groups were not significantly different before MCh challenge (Table 1). Before SS exposure on PND59, we found no significant difference in the R_L_ and C_dyn_ responses to MCh challenge between mice exposed to FA or SS in any of the three time periods (GD7, PND2, PND21; Figure 2). However, after SS exposure on PND59, the MCh dose–response curves were significantly elevated for R_L_ and significantly decreased for C_dyn_ in mice exposed to SS versus FA beginning on GD7 or on PND2 (Figure 3). For example, a 50 mg/mL MCh dose increased RL by 296% (95% CI, 222–370%) in the GD7 FA exposure group and by 544% (95% CI, 410–678%) in the GD7 SS exposure group (Figure 3). At the same time, 50 mg/mL MCh increased R_L_ by 315% (95% CI, 233–397%) in the PND2 FA exposure group and by 510% (95% CI, 402–618%) in the PND2 SS exposure group. The significant differences in the C_dyn_ response to MCh between SS- and FA-exposed mice were in the GD7 and PND2 exposure groups (95% CI, −90 to −62% vs. −57 to −35% and −84 to −60% vs. −54 to −42%, respectively). However, we found no significant difference in R_L_ or C_dyn_ responses to MCh between the PND21 FA- and SS-exposed groups. The same MCh dose produced a 320% (95% CI, 246–394%) increase in RL in the PND21 FA exposure group and 385% (95% CI, 301–471%) increase in the PND21 SS exposure group.

### Effect of SS during prenatal or postnatal period on changes of SP-immunoreactive NFD in tracheal smooth muscle

**Figure 4:**
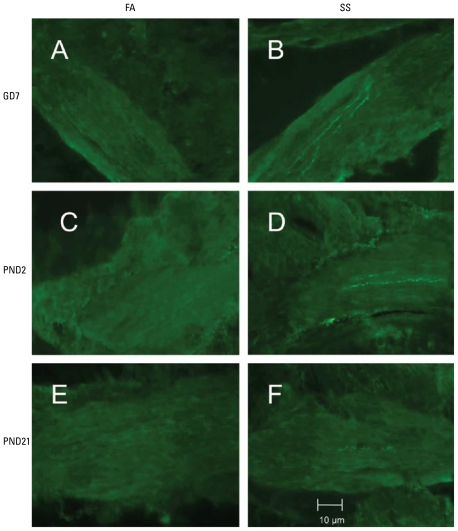
Fluorescence photomicrographs of SP-immunoreactive NFD within tracheal smooth muscle in GD7 FA- (*A*) or SS-exposed (*B*), PND2 FA- (*C*) or SS-exposed (*D*), and PND21 FA- (*E*) or SS-exposed (*F*) mice after SS exposure on PND59. (*A*, *C*, and *E*) FA exposure in GD7, PND2, and PND21 groups: negative SP immunoreactivity in tracheal smooth muscle. (*B* and *D*) SS exposure in GD7 and PND2 groups: increased SP-immunoreactive nerve fibers in tracheal smooth muscle. (*F*) SS exposure in PND21: few SP-immunoreactive nerve fibers are present in tracheal smooth muscle.

**Figure 5:**
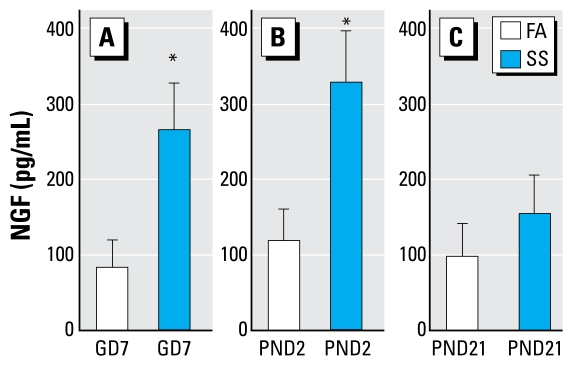
Changes of SP NFD in tracheal smooth muscle in GD7 (*A*), PND2 (*B*), and PND21 (*C*) FA- or SS-exposed mice after SS exposure on PND59. Data are mean ± SE of 8/group. *Significant difference between FA- and SS-exposed mice (*p* ≤ 0.05).

The percent area of SP-immunoreactive nerve fibers in tracheal smooth muscle was significantly different between FA- and SS-exposed mice in the GD7 and PND2 exposure groups. SP NFD in trachea was significantly increased in GD7 and PND2 exposure groups 16 hr after exposure to SS on PND59 (95% CIs, 0.04–0.105% and 0.034–0.078%, respectively, for FA-exposed mice; 0.08–0.2% and 0.09–0.19%, respectively, for SS-exposed mice) (Figures 4 and 5). At the same time, SP NFD did not change significantly in FA- and SS-exposed mice in PND21 groups, increasing only from 0.04–0.10% in PND21 FA-exposed mice to 0.06–0.12% in SS-exposed mice (Figures 4 and 5). These findings suggest that the initial exposure to SS during prenatal or early postnatal periods significantly enhance the levels of SP innervation in tracheal smooth muscle after SS exposure at PND59. However, the changes in SP NFD did not occur in SS- and FA-exposed mice in the PND21 group.

### Assessments of airway inflammation and NGF release from BALF

The total cells in the BALF were not markedly different between FA- and SS-exposed mice in the GD7 and PND2 exposure groups. However, the percentages of neutrophils were significantly higher in SS- versus FA-exposed mice in the GD7 (18.2% vs. 7.01%) and PND2 (17.84% vs. 8.27%) groups 16 hr after SS exposure on PND59 (Table 2). The percentage of neutrophils was not significantly different in SS- and FA-exposed mice in the PND21 group (12.84% vs. 8.27%; Table 2).

**Figure 6:**
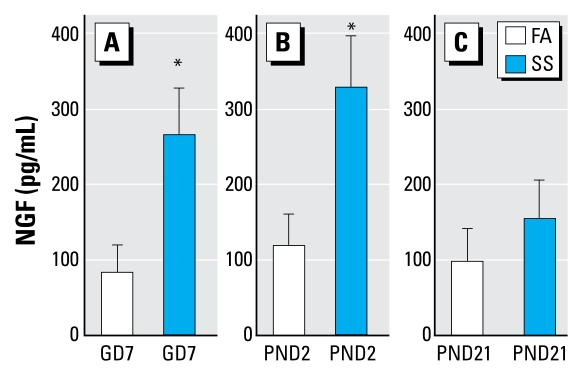
Levels of NGF release in BALF in GD7 (*A*), PND2 (*B*), and PND21 (*C*) FA- or SS-exposed mice after SS exposure on PND59. Data are mean ± SE of 6/group. NGF was measured by ELISA. *Significant difference between FA- and SS-exposed mice (*p* ≤ 0.05).

Thee concentrations of NGF in BALF were significantly higher in SS- versus FA-exposed mice in the GD7 and PND2 exposure groups 16 hr after SS exposure on PND59 (95% CI, 167–375 vs. 23–143 pg/mL and 214–450 vs. 58–180 pg/mL, respectively). At the same time, the level of NGF was not significantly different in SS versus FA mice in the PND21 group (95% CI, 70–234 vs. 24–166 pg/mL; Figure 6).

## Discussion

ETS exposure in infants and children increases respiratory symptoms such as coughs (Dodge 1982; Ekwo et al. 1983), wheezing (Dodge 1982), and airway obstruction (Sherrill et al. 1992; Wang et al. 1994). Maternal smoking during pregnancy also affects lung function in early life (Brown et al. 1995; Tager et al. 1995). Epidemiologic studies show that children are more susceptible to adverse respiratory effects of passive smoking than are adults (Lebowitz et al. 1992), suggesting that exposure to ETS in early development may be a predisposing factor for such conditions. The results obtained from the present study show that lung function and SP innervation in tracheal smooth muscle after exposure to SS on PND59 were significantly different and that the level of NGF in BALF was significantly elevated in mice initially exposed to SS versus FA during early periods of development (prenatal and early postnatal period). However, initial exposure to SS during a later period of development (PND21) did not appear to affect lung function or SP innervation of the airways after SS exposure on PND59, suggesting that a critical period of susceptibility exists during prenatal and early postnatal periods. The data also indicate that increased SP innervation is associated with elevated NGF levels in airways.

Some studies have shown that children exposed to smoke in early life have both immediate and persistent detrimental effects on lung health (Burchfiel et al. 1986; Chilmonczyk et al. 1993; Frischer et al. 1992; Martinez et al. 1992; Morgan and Martinez 1992; Ronchetti et al. 1992; Singh et al. 2003; Skorge et al. 2005; Tager et al. 1983; Weitzman et al. 1990). However, most of these studies focus on immune mechanisms that contribute to the development of airway diseases. Airway sensitivity and airway responsiveness change with age (Aberg and Alder 1973; Clerici et al. 1989; Duncan and Douglas 1985; Sparrow and Mitchell 1990). Innervation of the airways and lungs is not fully developed in the early postnatal stage of life. The sensitivity of airway nerves in newborns is higher than in adults (Larsen et al. 2004). Interestingly, the distribution and density of peptidergic nerves in young children are lower than in adults (Hislop et al. 1990). Thus, neurotransmitter expression is age related, which suggests the possibility that SS exposure during periods of rapid lung growth and remodeling may alter the normal developmental process. SP is localized within cell bodies of sensory neurons that project their peripheral axons to the respiratory system, innervating mucosal blood vessels, mucous glands, airway smooth muscle, and epithelium (Dey et al. 1990; Lee et al. 1985; Lundberg et al. 1983b, 1984). SP acts as a neuromodulator, increasing cholinergic sensitivity of airway smooth muscle (Cheung et al. 1994) and increasing the excitability of airway neurons (Myers et al. 1990). Transient increases in SP in sensory cell bodies projecting to the airways and in axons innervating the airway wall have been observed after irritant exposures (Graham et al. 2001; Hunter et al. 2000; Lundberg et al. 1983a; Wilfong and Dey 2004, 2005). Our findings in the present study, that an initial exposure to SS during prenatal and early postnatal periods in mice was associated with significantly decreased lung function and increased SP NFD in tracheal smooth muscle after exposure to SS on PND59, indicate that SP could modulate the pathogenesis of AHR later in life. A recent study found that ETS exposure during early life enhanced SP nerves in bronchial epithelium in infant monkeys, accompanied by inflammatory changes in the lung (Yu et al. 2008). Several studies have found that increased SP synthesis is involved in the pathogenesis of human airway diseases. Ollerenshaw et al. (1991) demonstrated increased SP NFD in airway smooth muscle of severe asthmatics. SP NFD was increased in airway epithelium from human subjects with persistent non productive cough (O’Connell et al. 1996). These studies show that enhanced SP innervation in human airway sensory nerves may contribute to altered airway function.

Neurotrophins, such as NGF, are essential in promoting and maintaining differentiation, growth, and survival of cells of the central and peripheral nervous systems, including those that innervate the airways (Leon et al. 1994; Levi-Montalcini 1987). Disruption of normal synthesis and release of neurotrophic factors changes airway innervation after inhalation of toxic material in adult lungs and leads to disease-related abnormalities in the respiratory system (Hu et al. 2002). Our recent studies found that exposure to toluene diisocyanate, a known airway irritant, directly enhances the level of NGF in airway (Wilfong and Dey 2004, 2005). We designed the present study to address the hypothesis that increased NGF after SS exposure enhances SP innervation in airways. Our data show that levels of NGF in BALF and SP innervation were both elevated in animals exposed to SS during prenatal or early postnatal periods, suggesting that NGF enhances SP innervation. We reported previously that NGF was involved in mediating increased SP expression in ferret airways after ozone exposure (Wu and Dey 2006), supporting the possibility that NGF serves as a signaling molecule during inflammatory events in the airways by regulating sensory neuropeptide production. Because NGF also stimulates neuronal growth, enhanced SP innervation of the airway wall may also occur in axon sprouting. Therefore, SS-enhanced NGF production in lung may produce increased SP levels in the airway both by increasing SP expression and by increasing the branching of SP-containing nerves in the airways. Increased NGF levels in BALF of infants after respiratory syncytial virus infection is associated with increased preprotachykinin gene expression in airways (Hu et al. 2002; Tortorolo et al. 2005), supporting the possibility that NGF contributes to altered SP expression in early life. Thus, the effects of SS-induced NGF release on airway SP innervation during prenatal and early postnatal life, when the airways are undergoing intense growth and development, might cause a change in airway responsiveness when exposed to SS much later in life.

Although we did not determine the exact mechanism of the enhanced MCh dose response by SS in the present study, results showed that the R_L_ and C_dyn_ responses to MCh challenge between FA- and SS-exposed mice in the three different groups were not significantly different before SS exposure on PND59 but were significantly altered in SS- versus FA-exposed mice in the GD7 and PND2 exposure groups after SS exposure on PND59. This alteration, in parallel with increased SP NFD in tracheal smooth muscle, indicates that SP may be involved. SP is a known bronchoconstrictor (Barnes et al. 1991), with direct actions on neurokinin 1 receptors present on airway smooth muscle. However, previous studies also showed that SP enhances cholinergic responsiveness either through a direct effect on sensitivity of airway smooth muscle (Tanaka and Grunstein 1990) or by enhancing acetylcholine release from parasympathetic nerve terminals (Larsen et al. 2004; Sekizawa et al. 1987).

The present study shows that an initial exposure to SS versus FA during the prenatal period was associated with significant changes in lung function and SP innervation in tracheal smooth muscle after exposure to SS on PND59. But the physiologic mechanisms that cause these changes during *in utero* exposure remain unclear. ETS is composed of almost 4,000 chemicals, including nicotine, carbon monoxide, cyanide, and ammonia. However, nicotine is probably the major component that crosses the placental barrier into the fetal circulation. Sekhon et al. (2001) showed that fetal monkeys exposed to nicotine in the pre-natal period had an increase in airway resistance. Chen et al. (2005) found that animals treated with nicotine *in utero* had a reduction in alveolar surface area used for gas exchange. A recent study showed that nicotine, acting through the nicotinic acetylcholine receptor, alters lung branching morphogenesis during the prenatal period (Wongtrakool et al. 2007). These studies indicate that nicotine potentially alters normal lung growth during the prenatal period, which may subsequently affect pulmonary function later in life. However, some other products from ETS exposure, such as 4-aminobiphenyl or nitrosamine 4-methyl-nitrosamino-3-pyridyl-1-butanone, can also cross the placenta, bind to fetal hemoglobin, and induce gene injury in the lung (Lackmann et al. 2000; Neri et al. 2006). It is also well known that the reduction of oxygen and the increase of carbon monoxide during ETS exposure are detrimental to lung development during the prenatal period (Buttigieg et al. 2008; California Environmental Protection Agency 2005). Because of the complexity of ETS exposure, we could not identify specific components involved in lung injury in our study.

In conclusion, exposure to SS during prenatal (maternal exposure) and early postnatal life was associated with changes in lung function later in life. These findings suggest that prenatal and early postnatal life are periods of susceptibility to inhaled SS that are associated with enhanced SP innervation of the airways and higher levels of NGF in the lung. Interestingly, we did not observe these responses when SS exposure occurred in later postnatal life (near puberty in mice), suggesting that the period of susceptibility is confined to the prenatal and early postnatal periods in mice.

## Figures and Tables

**Table 1:** The baseline of R_L_ (cm H_2_O/mL/sec) and C_dyn_ (mL/cm H_2_O) in different groups.

	Before SS exposure on PND59		After SS exposure on PND59	
Group	R_L_	C_dyn_	R_L_	C_dyn_
GD7 SS	1.12 ± 0.11	0.053 ± 0.014	1.18 ± 0.19	0.046 ± 0.016
GD7 FA	1.04 ± 0.13	0.058 ± 0.016	1.11 ± 0.11	0.054 ± 0.017
PND2 SS	1.01 ± 0.09	0.065 ± 0.011	1.09 ± 0.13	0.053 ± 0.012
PND2 FA	0.94 ± 0.12	0.069 ± 0.016	1.03 ± 0.15	0.059 ± 0.007
PND21 SS	1.08 ± 0.05	0.062 ± 0.008	1.02 ± 0.10	0.069 ± 0.011
PND21 FA	1.01 ± 0.08	0.067 ± 0.007	0.96 ± 0.12	0.072 ± 0.014

Data are mean ± SE of 6 mice/group.

**Table 2:** Effect of SS or FA exposure at GD7, PND2, and PND21 mice after SS exposure on PND59 on leuko-cyte counts in BALF.

Group	Total cells (×10^5^)	Neutrophils (%)	Eosinophils (%)	Lymphocytes (%)	Macrophages (%)
GD7 SS (*n* = 6)	39.32 ± 8.54	18.20 ± 3.61*	4.55 ± 1.83	5.62 ± 2.87	71.63 ± 7.92
GD7 FA (*n* = 6)	29.56 ± 7.93	7.01 ± 1.17	5.76 ± 2.19	6.28 ± 2.71	80.95 ± 5.60
PND2 SS (*n* = 6)	38.21 ± 6.51	17.84 ± 3.28*	3.27 ± 2.14	6.89 ± 2.89	72.00 ± 5.65
PND2 FA (*n* = 6)	34.62 ± 4.39	8.27 ± 2.54	3.87 ± 1.88	8.99 ± 3.54	78.87 ± 5.98
PND21 SS (*n* = 6)	43.45 ± 4.59	12.84 ± 4.70	3.51 ± 2.12	4.38 ± 1.39	79.27 ± 8.62
PND21 FA (*n* = 6)	36.98 ± 6.33	10.27 ± 3.37	4.85 ± 2.66	5.21 ± 2.62	79.67 ± 9.31

Data are mean ± SE.

* Significant difference in corresponding data between FA and SS groups (*p≤* 0.05).
